# Intrafemoral Injection of Human Hematopoietic Stem and Progenitor Cells into Immunocompromised Mice

**DOI:** 10.3791/66315

**Published:** 2023-12-08

**Authors:** Emily F. Calderbank, Laura Magnani, Elisa Laurenti

**Affiliations:** Wellcome and Medical Research Council Cambridge Stem Cell Institute, Department of Haematology, University of Cambridge, Cambridge, United Kingdom

## Abstract

Hematopoietic stem cells (HSCs) are defined by their lifelong ability to produce all blood cell types. This is operationally tested by transplanting cell populations containing HSCs into syngeneic or immunocompromised mice. The size and multilineage composition of the graft is then measured over time, usually by flow cytometry. Classically, a population containing HSCs is injected into the circulation of the animal, after which the HSCs home to the bone marrow, where they lodge and begin blood production. Alternatively, HSCs and/or progenitor cells (HSPCs) can be placed directly in the bone marrow cavity.

This paper describes a protocol of intrafemoral injection of human HSPCs into immunodeficient mice. In short, preconditioned mice are anesthetized, and a small hole is drilled through the knee into the femur using a needle. Using a smaller insulin needle, cells are then injected directly into the same conduit created by the first needle. This method of transplantation can be applied in varied experimental designs, using either mouse or human cells as donor cells. It has been most widely used for xenotransplantation, because in this context, it is thought to provide improved engraftment over intravenous injections, therefore improving statistical power and reducing the number of mice to be used.

## Introduction

Blood has one of the highest regeneration rates in the human body, producing 1 × 10^12^ cells per day in the adult human bone marrow^1^. Hematopoietic stem cells (HSCs) guarentee blood production over the lifespan by the process of hematopoiesis and are defined by their capacity to produce all blood cell types (multipotentiality) while maintaining themselves (self-renewal). Historically, the gold standard for testing the function of an HSC has always relied on transplantation, testing the ability of a donor population to reconstitute all blood lineages of a mouse long term (commonly defined as a minimum of 20 weeks)^2^. A large body of functional work spanning several decades has demonstrated that the HSC compartment is heterogeneous in both lineage output and long-term reconstitution. The toolkit to study hematopoiesis has expanded considerably over the years, with many new techniques, including *in vitro* single-cell functional assays, single-cell -omics approaches, and lineage tracing^3^. The latter have conclusively demonstrated that the contributions of HSC and multipotent progenitors largely differ in native hematopoiesis and under the stress imposed by transplantation. All these techniques complement transplantation assays, which remain a key assay to assess long-term repopulation capacity of HSCs. In the context of the study of human hematopoiesis, xenotransplantation provides the only method to experimentally assess self-renewal in a whole-organism setting.

Xenotransplantation of HSCs is commonly performed using intravenous injection of cells into immunocompromised mice. However, HSCs are rare^4^ and access to human samples containing HSCs is limited. In 2003, the group of John Dick adapted a protocol for bone marrow aspiration and intrafemorally injected non-obese diabetic/severe combined immunodeficiency (NOD-SCID) mice with Lin^−^CD34^+^ umbilical cord blood (CB) cells^5^. To our knowledge, there has been no reported formal comparison of intravenous vs intrafemoral injections long-term and serial transplantation outcomes. However compared directly with intravenous injections, intrafemoral injections provide larger graft sizes with the same number of transplanted cells^6^, at least in the short-term. In addition, engraftment can be detected with many fewer hematopoietic stem and progenitor cells (HSPCs) transplanted. This is thought to be because intrafemoral delivery bypasses the need for HSCs to home to the bone marrow, which in the xenograft context is limiting due to lack of cross-species reactivity for a number of receptors and cytokines. Via the use of intrafemoral injections, Notta and colleagues were the first to transplant single human HSCs^7^, though extra considerations need to be taken, as described in their methods. Intrafemoral delivery of HSPCs also has limitations. The injection itself disrupts and destroys part of the bone marrow, and therefore is not indicated for studies of the crosstalk between HSCs and their bone marrow microenvironment. Additionally, the maximum number of cells is limited by the volume of that bone cavity and that may be too few for some applications. As with every technique, its application in a specific experiment needs to be weighed up based on the benefits/disadvantages and the question being asked. In the context of xenotransplantation, if the aim of the experiment is to test engraftment a low number of human HSPCs with no assessment of microenvironment, intrafemoral delivery is usually preferred over intravenous injection.

## Protocol

All animal research presented here adheres to the Animals (Scientific Procedures) Act 1986 Amendment Regulations 2012 and was performed after ethical review and approval by the University of Cambridge Animal Welfare and Ethical Review Body (AWERB). Female NOD.Cg-Prkdc^scid^Il2rg^tm1Wjl^/SzJ (NSG) mice, aged between 12 and 16 weeks (~21–30 g), bred in-house and maintained in a Specific-Pathogen-Free animal facility, were used for intrafemoral injections. De-identified CB samples were collected from healthy donors after informed consent by the Cambridge Blood and Stem Cell Biobank (CBSB) in accordance with regulated procedures approved by the Cambridgeshire Local Research Ethics Committee (18/EE/0199). De-identified CB samples were obtained with informed consent from healthy donors through the Cambridge Blood and Stem Cell Biobank (CBSB) in accordance with regulated procedures approved by the Cambridgeshire Local Research Ethics Committee (18/EE/0199 research study).

### 1 Preparation of the mouse

1.1. Before irradiation, notch the ears of the mice for identification and weigh them for baseline weight.

1.2. Twenty-four hours before transplantation of the cells, sub-lethally irradiate the mice with 2.4 Gy radiation.

### 2 Preparation of cells

NOTE: For these injections, cells can be used from fresh or frozen samples. Specific subpopulations of cells can be sorted by flow cytometry. Alternatively, cells can be cultured in the desired conditions before transplantation. For the experiments shown, we are using frozen CB CD34^+^ cells.

2.1. Thaw the cells in a 50 mL tube by the dropwise addition of 10x the cell’s volume of prewarmed IMDM + 50 % Fetal Bovine Serum (FBS) + 0.1 mg/mL DNase while agitating the tube manually. Centrifuge the cells at 500 × *g* for 5 min.

2.2. Resuspend the cells in 20–40 μL (ideally 25 μL) of PBS + Penicillin-Streptomycin (10 U/mL or 0.1 %) per mouse. If possible, allow extra cells for a dead volume when taking up the cells into the syringe for injections. Store the cells on ice until injections. The maximum number of cells per injection is 4 million cells.

### 3 Preparation for intrafemoral injection in the animal facility

3.1. Prepare the following three needles required for this procedure:

3.1.1. Prepare a 3 mL syringe with a 27 G 1/2″ needle.

3.1.2. Prepare a 0.5 mL Insulin Syringe with 29 G x 12.7 mm needle containing the cell suspension.

3.1.3. Prepare a 1 mL Insulin Syringe with 29 G x 0.5” needle with 0.1 mg/kg buprenorphine (100 μL).

3.2. Anesthetize the mice in an anesthetic box (through the inhalation of isoflurane 2% (v/v) and oxygen 2 L/min). Transfer to a nose cone and confirm its readiness for the procedure by toe pinch.

NOTE: This procedure is carried out in a containment level 2 biosafety hood and sterile conditions are used.

### 4 Intrafemoral injection

4.1. Lay the mouse on its back and have its hind leg flexed. Secure the hind leg using the non-dominant hand by placing the thumb on the foot, the middle finger on the hip, and the index finger on the outside of the femur.

4.2. Gently shave or pluck the hair from around the kneecap and use an alcohol swab to wipe down the area.

4.3. Use the 3 mL syringe with a 27 G 1/2” needle and aim for the top inner corner of the kneecap to gently drill a hole through the skin towards the femur. Although the needle may be rotated back and forth at first, only use a clockwise motion once into the bone, until the whole needle is in the bone.

NOTE: The goal of this step is to generate a conduit for cell delivery. (Optional) To check if the needle is correctly in the bone, release the leg and rotate the syringe side to side; if it is correctly in the bone, the whole leg should move with the rotation. Re-secure the leg using the non-dominant hand by placing the thumb on the foot, the middle finger on the hip and the index finger on the outside of the femur, before continuing.

4.4. Remove the needle using an anticlockwise rotation until it is halfway out. At this point, use an alcohol swab to wipe around the needle (there may be a small drop of blood) and then continue to turn the needle anticlockwise and remove the needle.

4.5. Insert the 0.5 mL insulin syringe containing the cells in the femoral shaft via the same conduit. Once the needle is in, make note of feeling a scratch indicating correct location. At this point, release the grip on the leg, but be careful not to remove the needle from the leg. Then, gently, inject the 25 μL of cell suspension into the femur and remove the needle.

NOTE: The ‘scratch’ described is like hitting a rough surface and if the needle feels it has gone in smoothly with no rough feeling, it is not in the correct place. Most users understand this clearly once they are successful in their practice.

### 5 Post injection care

5.1. Inject the mouse subcutaneously with Buprenorphine (0.1 mg/kg, 100 μL) before returning it to its cage and monitoring for recovery from the anesthetic.

NOTE: The mouse is not left unattended until it has regained sufficient consciousness to maintain sternal recumbency and not returned to the company of other animals until fully recovered.

5.2. Mice are unlikely to show any adverse effects to the intrafemoral injection; however, intrafemoral injection may result in reduced activity and pain in the operated area. Assess swelling of the hock, pain, and mobility following the procedure; mice should regain normal limb mobility within 24 h of injection.

### 6 Analyzing the data

6.1. Sacrifice the mice by cervical dislocation or any approved method post injection at any time e.g., 24–48 h for homing experiments, 4–12 weeks for short-term HSC activity, >18 weeks for long-term engraftment. If the analysis of peripheral blood (PB) is planned, mice must be bled before sacrifice by any authorized method, yielding ideally 50-100 μL of blood (no more than 10% of total volume). Remove rear legs bones and spleen according to standard dissection protocols.

6.2. For bone marrow: keep the injected femur (IF), the non-injected bones (rear leg tibias and non-injected femurs, termed BM) separate. Flush the bones using 1 mL IMDM + 5 % FBS and a 36 G x ¾” needle then centrifuge 5 min 500 x g. Resuspend in 500 μL PBS + 3 % FBS and then take 50 μL to a well of a 96 round-bottom plate for staining. Remaining bone marrow can be frozen down and stored in liquid nitrogen for further *ex vivo* or secondary *in vivo* experiments.

6.3. For the PB: wash out the collection tube with 100 μL PBS + 3 % FBS and transfer the blood to a 5 mL FACS tube, further wash out with 100 μL PBS + 3 % FBS and transfer to the FACS tube. Make up to 2.5 mL with PBS + 3 % FBS. Carefully pipette 1 mL of Pancoll to the bottom of the FACS tubes. Centrifuge 25 min 500 x *g*, brake off. Collect the buffy coat layer and transfer to 1.5 mL tubes, top up to 1.5 mL with PBS + 3 % FBS. Centrifuge 5 min 500 x *g*. Resuspend in 50 μL PBS + 3 % FBS and transfer to a well of a 96 round-bottom plate for staining.

6.4. For the spleen: Place the spleen in a cell strainer placed on a 50 mL tube and crush with the plunger from a 3 mL syringe. 1 mL at a time add 5 mL of IMDM + 5 % FBS to wash the cells through. Take 50 μL to a well of a 96 round-bottom plate for staining.

6.5. Prepare an antibody mastermix: here is an example for 10 samples: aliquot 550 μL of PBS + 3 % FBS (50 μL / sample + 10 % extra) in an 1.5 or 2 mL tube. Add individual antibodies so that their final concentration is 2X based on their titration (for example for an antibody titrated 1:100, here 11 μL would be added). Choose an antibody panel that assesses potential differentiation across all blood lineages of interest, for example CD19/FITC, GlyA/PE, CD45/PECy5, CD14/PECy7, CD33/APC, CD19/Alexa 700, CD45/BV510, and CD3/APCCy7.

6.6. Staining: add 50 μL of antibody mastermix to each of the wells of the 96 round-bottom plate containing the samples to stain. Stain for 20 min at room temperature then add 100 μL PBS + 3 % FBS to wash the cells and centrifuge 5 min 500 x *g*. Remove the supernatant.

6.7. Resuspend in 200 μL PBS + 3 % FBS and pass each sample through a FACS tube with a cell strainer cap. To bone marrow and spleen samples add a further 200 μL PBS + 3 % FBS.

6.8. Flow cytometry controls: Make single stain controls using compensation beads by adding 100 μL PBS, 1 drop of positive beads, 1 drop of negative beads and 1 μL of the antibody to a FACS tubes. Leave for 5 min at room temperature and then add 300 μL PBS. Take 50 μL of unstained bone marrow cells made up to 400 μL PBS + 3 % FBS as an unstained control.

6.9. Samples can be analyzed by flow cytometry on a BD LSR Fortessa X-20 Cell Analyzer or equivalent. First run single stain controls and unstained for compensation as advised for the cytometer used. Then set up gating as in Figure 3A. For PB, run all cells and for bone marrow and spleen run minimum 50,000 events per sample depending on human engraftment levels.

## Representative Results

**Figure 1:**
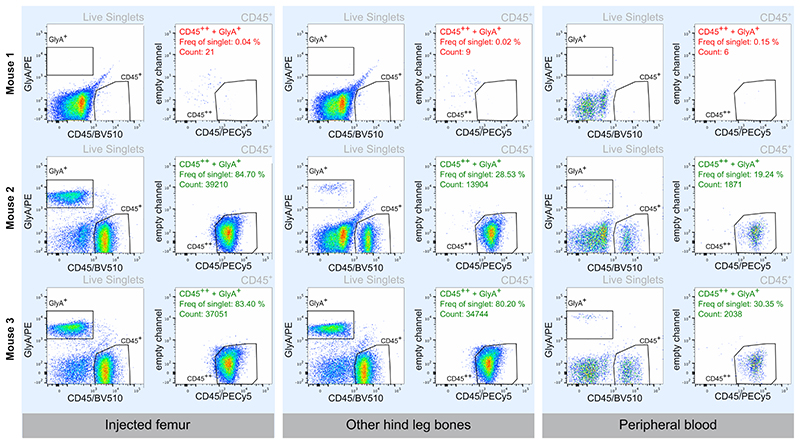
Representative human engraftment in the injected femur (IF), other hind leg bones (BM), and peripheral blood of mice intrafemorally injected with human hematopoietic stem and progenitor cells. Representative flow plots of non-engrafted (Mouse 1) and engrafted (Mice 2 and 3) mice. Mice are counted as engrafted when (% CD45^++^ + % GlyA^+^) ≥ 0.01% and at minimum of 30 cells were recorded in both gates. Mouse 2 demonstrates a case where the engraftment in the BMis lower than the IF. Mice were transplanted with CB CD34^+^ HSPCs cells and tissues were analyzed at 12 weeks.

**Figure 2:**
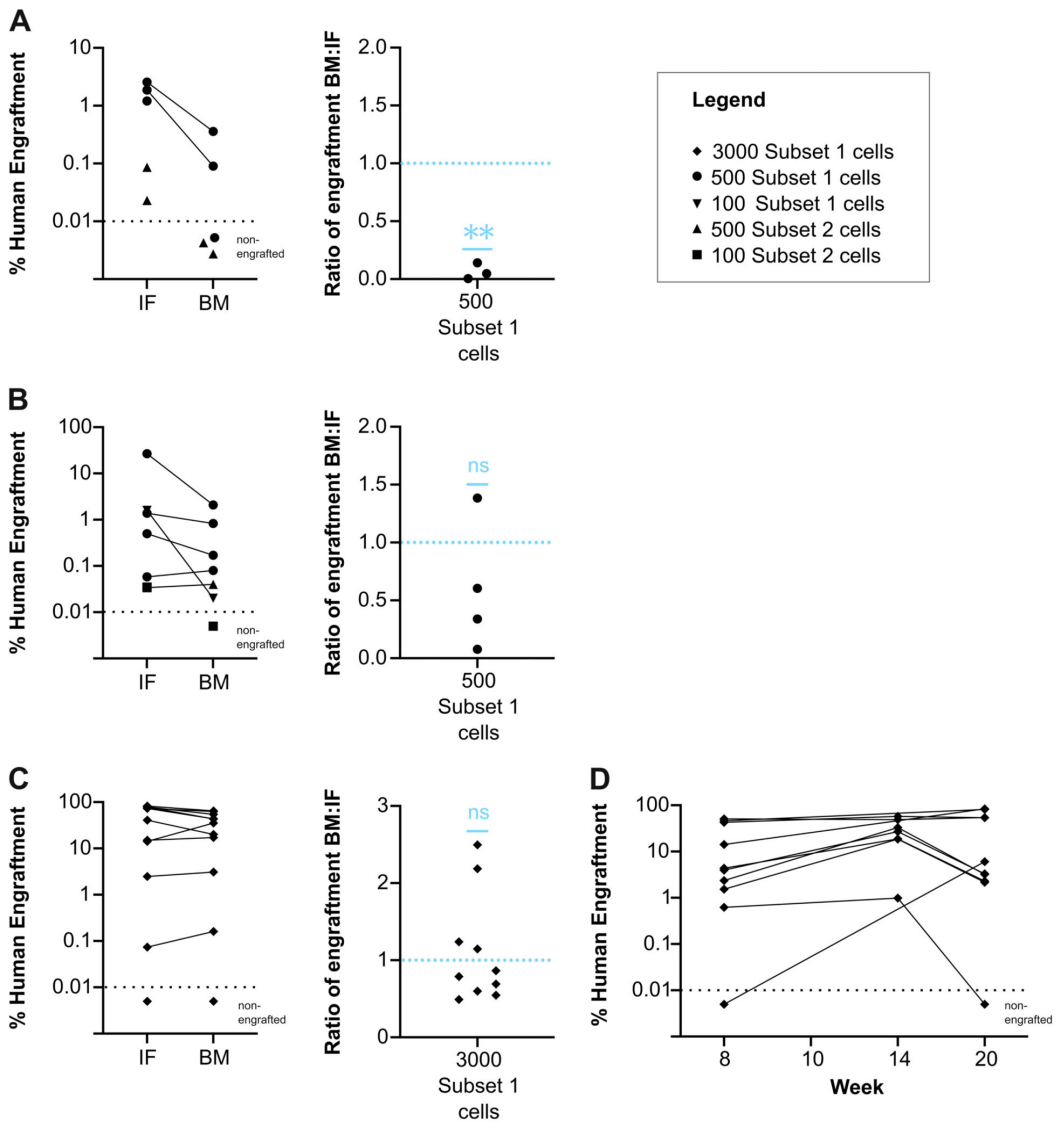
Comparison of engraftment in the injected femur and other hind leg bones at 4-, 8-, and 20-weeks post intrafemoral injection. **A-C):** Size of the human graft (% CD45^++^ + % GlyA^+^) in IF and BM (left panels) and the ratio of engraftment in BM:IF (right panels) at 4 weeks (**A**, n= 5 mice), 8 weeks (**B**, n=7 mice) and 20 weeks (**C**, n=11 mice) post transplantation. Mice were engrafted with 100, 500 or 3000 Subset 1 (CD19^−^CD34^+^CD38^−^CD45RA^−^CD34^lo^CLEC9A^hi^) or Subset 2 (CD19^−^CD34^+^CD38^−^CD45RA^−^CD34^hi^CLEC9A^lo^) cells. Cell type and dose depicted by different symbols (see legend). Mice shown below the black dashed line are considered non engrafted as their graft size falls below the threshold ((% CD45^++^ + % GlyA^+^) ≥ 0.01 % and at least 30 cells recorded). CD45^++^ cells positive for two distinct CD45 antibodies. Ratio of engraftment BM:IF for high dose mice only, 500 Subset 1 cells (A n=3, B n=4) or 3000 Subset 1 cells (C n=10). **p<0.01 by one-sample t-test. **(D):** Percentage human engraftment (% CD45^++^ + % GlyA^+^) in PB across time. Mice were engrafted with 3000 Subset 1 (D, n=10 mice). All data is a re-analysis of the data published in Belluschi *et al*^8^. Abbreviations: IF = intrafemoral; BM = bone marrow (other hind leg bones), PB = peripheral blood.

The engraftment of the intrafemorally injected cells can be assessed at any time point from 24 h onwards depending on the experimental design. At the end time point, IF, BM, PB, and spleens may be collected. These can be processed, and the level of engraftment assessed via flow cytometry. To robustly call human engraftment even at low levels, we stained with two distinct antibodies against human CD45 (clone HI30 and clone 2D1). Only cells positive for both antibodies (CD45^++^) were considered of human origin. Threshold of engraftment were set as follows: (% CD45^++^ + % GlyA^+^) ≥ 0.01 % and a minimum of 30 cells recorded in both gates (Figure 1)^7,8^. Migration of human cells to bones other than the one injected is usually delayed. Therefore, if the bones are harvested before 8 weeks, it is likely that the engraftment of the IF is higher than the BM (Figure 2A). This tends to equalize after 8 weeks (Figure 2B-C). Engraftment levels in PB are expected to be variable (Figure 2D).

**Figure 3:**
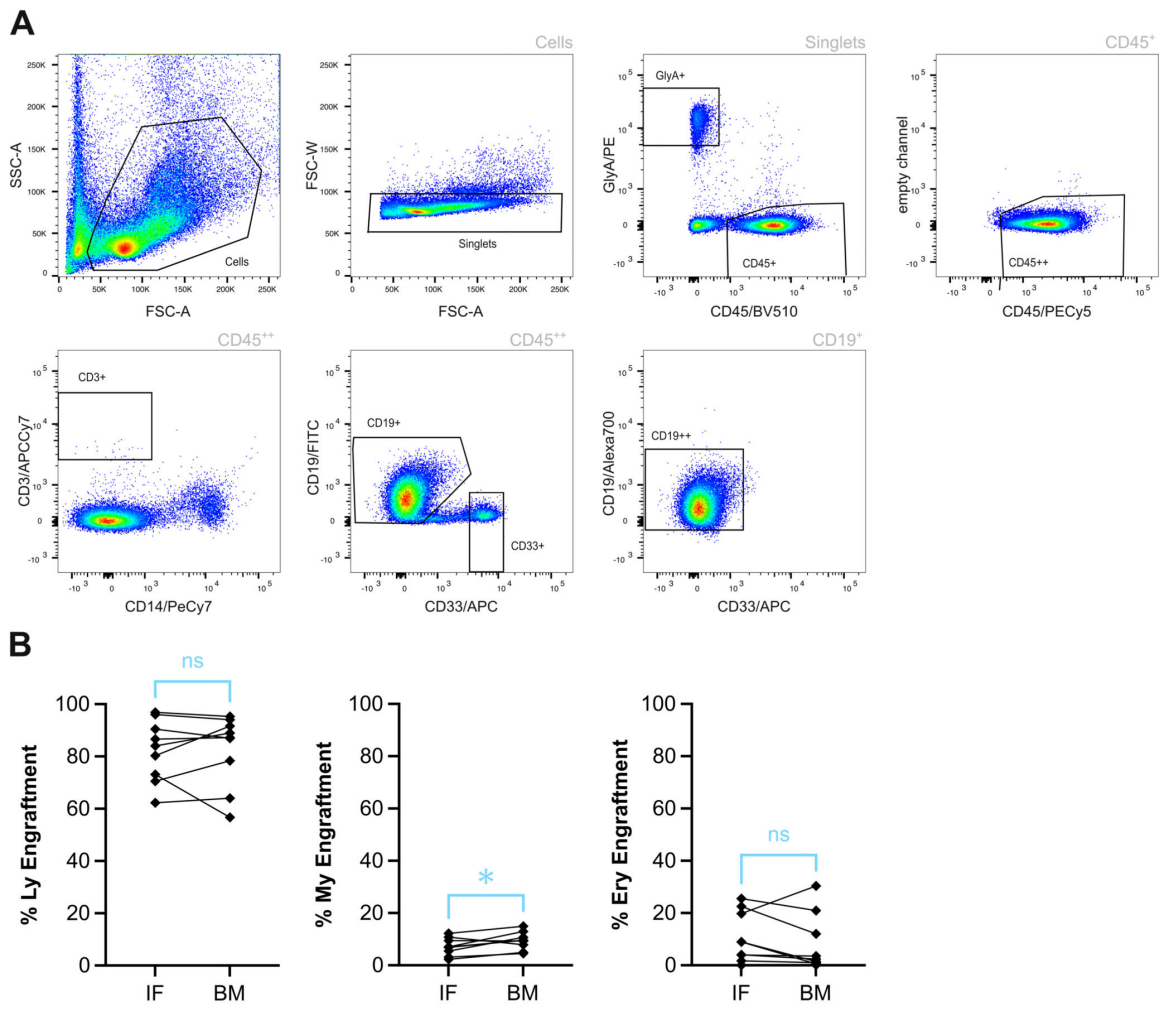
Analysis of lineage potential *in vivo*. Representative example of the gating strategy used to assess lineage potential *in vivo*. My lineage: CD45^++^ CD33^+^ ≥ 20 cells; Ly lineage: CD45^++^ CD19^++^ (positive for two distinct CD19 antibodies) and/or CD45^++^ CD3^+^ ≥ 20 cells; Ery lineage: CD45^−^GlyA^+^ ≥ 20 cells (A). Percentage of human Ly, My and Ery engraftment in the injected femur and other hind leg bones 20 weeks post transplantation of mice engrafted with 3000 Subset 1 (CD19^−^CD34^+^CD38^−^CD45RA^−^CD34^lo^CLEC9A^hi^) (B). Only mice with percentage human engraftment (% CD45^++^ + % GlyA^+^) > 1 % shown. *p<0.05 by paired t-test. This is a re-analysis of the data published in Belluschi *et al*^8^. Abbreviations: My = myeloid; Ly = lymphoid; Ery = erythroid; FSC-A = forward scatter area; FSC-W = forward scatter width; PE = Phycoerythrin; APC = Allophycocyanin; IF = intrafemoral; BM = bone marrow (other hind leg bones).

All bone marrow cells were stained using an antibody panel that assesses the level of engraftment across different blood lineages: CD19/FITC, GlyA/PE, CD45/PECy5, CD14/PECy7, CD33/APC, CD19/Alexa 700, CD45/BV510, and CD3/APCCy7. To assess which blood lineages are present in the human graft, we used the following thresholds: Lymphoid (Ly) lineage: CD45^++^ CD19^++^ (positive for two different CD19 antibodies) and/or CD45^++^ CD3^+^ ≥ 20 cells; Myeloid (My) lineage: CD45^++^ CD33^+^ ≥ 20 cells; Erythroid (Ery) lineage: CD45^−^ GlyA^+^ ≥ 20 cells (Figure 3A). Lineage composition is usually similar between IF and BM, but in certain instances, mature myeloid cells can be slightly but significantly increased in BM compared to IF (Figure 3B).

## Discussion

Intrafemoral injections are a useful tool in xenotransplantation when only a small number of HSPCs are available, providing improved engraftment compared to intravenous injections. However, the technique requires dexterity and training. When practicing, we would recommend using fresh cadavers of the correct weight range (see below) and injecting a colored dye (such as trypan blue) so that upon dissection, it is clear if the injection went into the femur and was restricted to it (no dye should be observed in the muscles). For this procedure, there are two critical steps: positioning and feeling the correct insertion of the second needle in the conduit. For positioning, although a method is recommended here, it is up to the individual to optimize how the mouse leg is most comfortably and reproducibly held. For example, a member of our team prefers to secure the leg from across the body. The key in any circumstance is that the leg, and therefore femur, is secure. The ‘scratch’ described to confirm whether the second needle is correctly inserted in the femur, is like hitting a rough surface but is difficult to explain further in writing. It is usually felt very clearly by most users once they are successful in their practice.

In the context of transplantation of human HSCs, and following a comparison of four immunocompromised mice, NSG mice are most commonly used for this technique^9^. Nonetheless since the publication of that article in 2010, other immunocompromised mice have been generated that can be utilized with intrafemoral injections such as NOD.*CgPrkdc^scid^Il2rg^tm1Wjl^*Tg(CMVIL3,CSF2,KITLG)1Eav/MloySzJ (NSG-SGM3)^10^ and *CSF1*^h/h^ *IL3/CSF2*^h/h^*SIRPA*^tg^ *THPO*^h/h^ *Rag2*^−/−^ *Il2rg*^−/^ (MISTRG)^11^, both producing human cytokines to support human hematopoiesis in mice. Alternatively, the need for irradiation may be overcome by the use of NOD,B6.SCID *Il2rγ^−/−^Kit^W41/W41^* (NBSGW) mice^12^.

This method does come with its limitations and considerations. As mentioned in the introduction, this method is not useful if the microenvironment of the bone is of interest to the research as it is damaged in the process of irradiation and injection. Additionally, the lineage output of the cells can vary depending on the mouse model. For NSG mice, an improvement of erythroid engraftment can be achieved if the mice are treated with human erythropoietin^8^. The age and sex of the mice must also be considered. If the mice are too young and therefore small, there is a risk of breaking the bone during the procedure and if the mice are older and bigger, it can be hard to drill the initial hole. We recommend mice between 21 g and 30 g in weight. Furthermore, as it has been published that for NSG mice, engraftment is improved in female mice compared to males^13^, we preferentially use females over males. Due to the potentially higher engraftment observed after intrafemoral injections compared to intravenous injections, the number of mice needed to obtain detectable measurements and statistically significant results may be lower. Caution should be taken with cell doses used, as high doses (>5 × 10^4^ CD34^+^ cells from CB) may lead to saturation of the injected bone at the expense of systemic engraftment^14^.

To date, our laboratory has used intrafemoral injections for the xenotransplantation of healthy CB^8^, spleen and PB^15^ cells. However, many other studies have shown their use can be beneficial for the transplantation of hematological samples from patients suffering from various hematological diseases. For example, Medyouf and colleagues found that the addition of patient-derived mesenchymal stromal cells improved engraftment of CD34^+^ cells from patients with Myelodysplastic syndromes after intrafemoral injection^16^. Intrafemoral injections are also commonly employed to test leukemic engraftment of Acute Myeloid Leukemia^17,18^ and Acute Lymphoblastic Leukemia samples^19,20^. Finally, intrafemoral injections have been used in a recent clinical trial of hematopoietic stem cell gene therapy of adult and pediatric patients affected by transfusion-dependent ß-thalassemia^21^, demonstrating their clinical relevance.

## Supplementary Material

Materials table

## Summary

Intrafemoral injections allow for the engraftment of a small number of hematopoietic stem and progenitor cells (HSPCs), by placing the cells directly in the bone marrow cavity. Here we describe an experimental protocol of intrafemoral injection of human HSPCs into immunodeficient mice.
